# Supplementary material for: Voluntary Physical Exercise Reduces Motor Dysfunction and Hampers Tumor Cell Proliferation in a Mouse Model of Glioma
Source: Int J Environ Res Public Health. 2020 Aug 5;17(16):5667. doi: 10.3390/ijerph17165667 (PMC7460183; doi:10.3390/ijerph17165667)
Supplement: Supplementary file 1 [file ijerph-17-05667-s001.pdf]

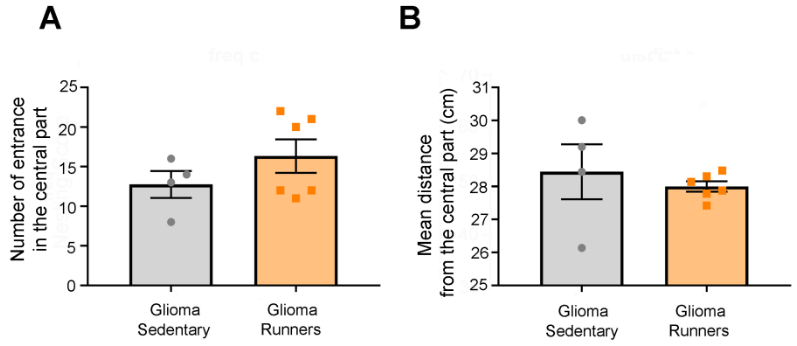

Figure S1. (A) Number of entrance in the central part. **(B).** Mean distance from the central part. Running glioma-bearing mice displayed the same anxiety level of sedentary glioma-bearing animals. Runner glioma mice (n = 6) didn't show any differences in the number of entrance and in the mean distance travelled from the central part of the arena with respect to the sedentary group (n = 4; t-test, p = 0.537 and p = 0.26). Data are expressed as mean ± SEM.

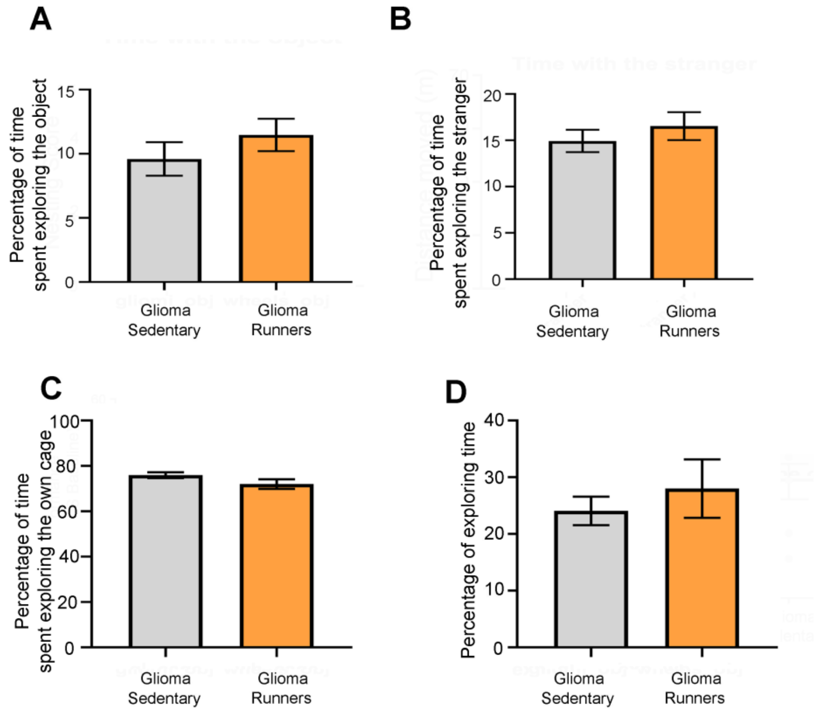

Figure S2: Sociability of running and sedentary glioma-bearing mice. Runner glioma mice (n = 6) didn't show any differences in the four parameters considered to evaluate the three chambers sociability test with respect to the sedentary group (n = 4). (**A**): t-test, p = 0.39; (**B**): t-test, p = 0.517; (**C**): t-test, p = 0.16; (**D**): t-test, p = 0.12). Data are expressed as mean ± SEM.
